# Double-edged sword effect of platelets in COVID-19

**DOI:** 10.1590/1677-5449.202201012

**Published:** 2023-03-06

**Authors:** Zohreh Jadali

**Affiliations:** 1 Tehran University of Medical Sciences, School of Public Health, Department of Immunology, Tehran, Iran.

Dear Editor,

With interest we read the article by Sobreira et al. about the thromboembolic complications of COVID-19 vaccines.^1^ This study has important implications, but no mention is made regarding these complications in COVID-19 patients. It is therefore necessary to discuss this topic and possible pathogenic mechanisms.

Coagulopathy is a common feature of severe acute respiratory syndrome coronavirus 2 (SARS-CoV-2) and its incidence increases in severe cases.^2^

The mechanisms of thrombotic events are multifactorial and platelets play a major role in this phenomenon. Beyond hemostasis and thrombosis, platelets are also capable of sensing and responding to invading pathogen and immune signals.^3^ Virus-platelet interactions may serve as part of the immune response or of viral counterdefense strategies. Platelets can interact with and respond to viruses via different mechanisms including phagocytosis and production of antiviral molecules. Conversely, they can also shelter several viruses and increase their transport ability throughout the circulation. Virus-mediated activation of platelets may also activate release of various pro-inflammatory mediators which lead to development of virus-induced immunopathology.^4^

Both positive and negative effects depend on the interaction between viral proteins and host cell receptors. Interactions can occur directly via various immune receptors in platelets or indirectly through plasma proteins. Platelets also express angiotensin-converting enzyme 2 (ACE2) which serves as the primary receptor for SARS-CoV-2 and facilitates virus entry into host cells.^5^

COVID-19 thrombotic complications may be the result of direct or indirect impacts of viral infection. SARS-CoV-2 can directly activate ACE2 and potentiates platelet activation. Moreover, the SARS-CoV-2 spike protein enhances the potential of thrombosis and recombinant human ACE-2 protein can suppress virus-induced platelet activation. The virus can also directly induce platelet releasing coagulation factors and inflammatory cytokines and increases formation of leukocyte-platelet aggregates.

Platelets may also be activated indirectly, through sensing of an inflammatory microenvironment and subsequent dysfunction of vascular endothelium, which are induced by viral infection. The inflammatory milieu may cause uncontrolled platelet activation which consecutively may lead to pathophysiological effector activities. Moreover, immune complex containing viral particles may impact on the platelet hyperactivity in COVID19 patients.^6^

Altogether, platelets may be affected by SARS-CoV-2. Therefore, understanding of the underlying mechanisms can be beneficial in promoting assessment and treatment of COVID-19 patients.
