# Supplementary material for: Double-edged sword effect of platelets in COVID-19
Source: J Vasc Bras. 2023 Mar 6;22:e20220101. doi: 10.1590/1677-5449.202201012 (PMC10010266; doi:10.1590/1677-5449.202201012)
Supplement: Related texts

# Reply: Covid-19 vaccines and thromboembolic complications

**Authors:** Marcone Lima Sobreira, Eduardo Ramacciotti, Adilson Ferraz Paschôa, Marcelo Fernando Matielo, Ivan Benaduce Casella, Guilherme Yazbek, Raphael de Athayde Soares, Bonno van Bellen, Marcos Arêas Marques

**Affiliations:** 1 Universidade Estadual Paulista “Júlio de Mesquita Filho” – UNESP, Botucatu, SP, Brasil.; 2 Loyola University Chicago, Chicago, IL, EUA.; 3 Universidade Estadual de Campinas – UNICAMP, Campinas, SP, Brasil.; 4 Universidade de São Paulo – USP, São Paulo, SP, Brasil.; 5 Hospital do Servidor Público Estadual de São Paulo, São Paulo, SP, Brasil.; 6 Real e Benemérita Associação Portuguesa de Beneficência de São Paulo, São Paulo, SP, Brasil.; 7 Universidade Federal do Estado do Rio de Janeiro – UNIRIO, Rio de Janeiro, RJ, Brasil.; 8 Universidade do Estado do Rio de Janeiro – UERJ, Rio de Janeiro, RJ, Brasil.

Dear Editor,

We would like to thank you for the important points on the topic of our article about thromboembolic complications of COVID-19 vaccines, which are quite relevant and help update knowledge on the issue.

The interactions described in relation to the pathophysiological role of platelets in activating the alleged potential thrombotic trigger are initiated by vaccine-induced thrombocytopenia, as occurs with thrombotic predisposition accompanying infection caused by COVID19.

Much remains to be discovered about the pathophysiology of COVID-19 infection, particularly the potential predisposition to thrombotic events. Thank you for sharing this data about complex platelet mechanisms and interactions that enhances the content of our article and helps us to understand and clarify the best assessment and treatment of COVID-19 patients.

Covid-19 vaccines and thromboembolic complications
